# Economic evaluation of Optilume, a drug‐coated balloon for recurrent anterior male urethral stricture

**DOI:** 10.1002/bco2.241

**Published:** 2023-04-10

**Authors:** Laura Kelly, Judith Shore, James Wright, Craig Patrick, Hayden Holmes

**Affiliations:** ^1^ York Health Economics Consortium, Enterprise House, Innovation Way University of York York UK; ^2^ Laborie Medical Technologies CORP Portsmouth New Hampshire USA

**Keywords:** andrology, economic evaluation, stricture, urethroplasty, urology

## Abstract

**Objectives:**

We aim to conduct an economic evaluation of the Optilume urethral drug‐coated balloon (DCB) compared with endoscopic management for the treatment of recurrent anterior male urethral stricture in England.

**Patients and Methods:**

A cohort Markov model was developed to estimate the costs and savings to the NHS over a 5‐year time horizon of adopting Optilume for the treatment of anterior urethral male stricture versus current endoscopic standard of care. A scenario analysis was conducted which compared Optilume to urethroplasty. Probabilistic and deterministic sensitivity analyses were performed to estimate the impact of uncertainties in model parameters.

**Results:**

When compared with current endoscopic standard of care Optilume resulted in an estimated cost saving of £2502 per patient if introduced in the NHS for the treatment of recurrent anterior male urethral stricture. In the scenario analysis, the use of Optilume compared with urethroplasty resulted in an estimated cost saving of £243. Results were robust to changes in individual input parameters as demonstrated in the deterministic sensitivity analyses, with the monthly probability of symptom recurrence associated with endoscopic management the only exception. Probabilistic sensitivity analysis results demonstrated that Optilume was cost saving in 93.4% of model iterations, when running 1000 iterations.

**Conclusion:**

Our analysis suggests that the Optilume urethral DCB treatment can be a cost‐saving alternative management option for the treatment of recurrent anterior male urethral stricture within the NHS in England.

## 1 BACKGROUND

A urethral stricture is the narrowing of the urethra, the tube that carries urine out of the body.^1^ Urethral strictures have multiple aetiologies, including an infection that leads to urethral inflammation and trauma to the urethra as a result of injury. A urethral stricture can occur to any individual; however, they are most likely to occur in males with the mean age being 59.^2^ In the year 2018/2019, according to NHS statistics, 16 185 men were admitted to the hospital in England for the treatment of a male urethral stricture.^2^

The first line treatment in England for anterior male urethral stricture is either urethrotomy or urethral dilation, also known as endoscopic management.^3^ Urethroplasty is a potential second‐line treatment for male urethral stricture in England. A urethroplasty is the reconstruction of the urethra via plastic surgery. Although urethroplasties are becoming more common in England, there is still a limitation on their availability to patients because the procedure requires a specialist centre with a trained urethroplasty surgeon.

Recurrence of male urethral stricture after receiving endoscopic treatment is common, reported to be as high as 16% after 1 month.^4^, ^5^ Recurrences of a urethral stricture often require further treatment, resulting in increased costs to the health system and impacting on patient's quality of life.^6^ Optilume was designed for the treatment of anterior male urethral strictures for people aged 18 years or older for stricture length equal to, or less than, 3 cm. Optilume is a surgical intervention that has a lower male urethral stricture recurrence rate than endoscopic management.^4^ This is the first analysis that attempts to estimate the cost impact of implementing Optilume as an alternative treatment for male urethral stricture in the England.

### 1.1 Objective

The aim of this study was to conduct an economic evaluation of the Optilume urethral drug‐coated balloon (DCB) compared with endoscopic management for the treatment of recurrent anterior male urethral stricture in England.

## 2 METHODS

### 2.1 Markov model

A cohort Markov model was developed in Microsoft Excel to estimate the cost impact of Optilume compared with endoscopic management in people with anterior male urethral stricture. Endoscopic management included urethral dilation (the use of a urethral dilation balloon without paclitaxel or urethral sounds) and urethrotomy (‘DVIU’, the use of a steel blade mounted on a urethroscope). The model was constructed based on the perspective of the National Health Service (NHS) in England and Personal Social Services (PSS). The model included a hypothetical cohort of male patients aged 59. A 5‐year time horizon was used. Five years was considered appropriate because it captured the benefits and costs associated with introducing Optilume whilst maintaining an acceptable level of uncertainty in the model given the paucity of longer term data. The cycle length was 1 month, and a 3.5% discount on costs was used. The primary model outcome was the incremental cost difference between Optilume and the comparator. Health‐related quality of life estimates were not included in the model.

**FIGURE 1:**
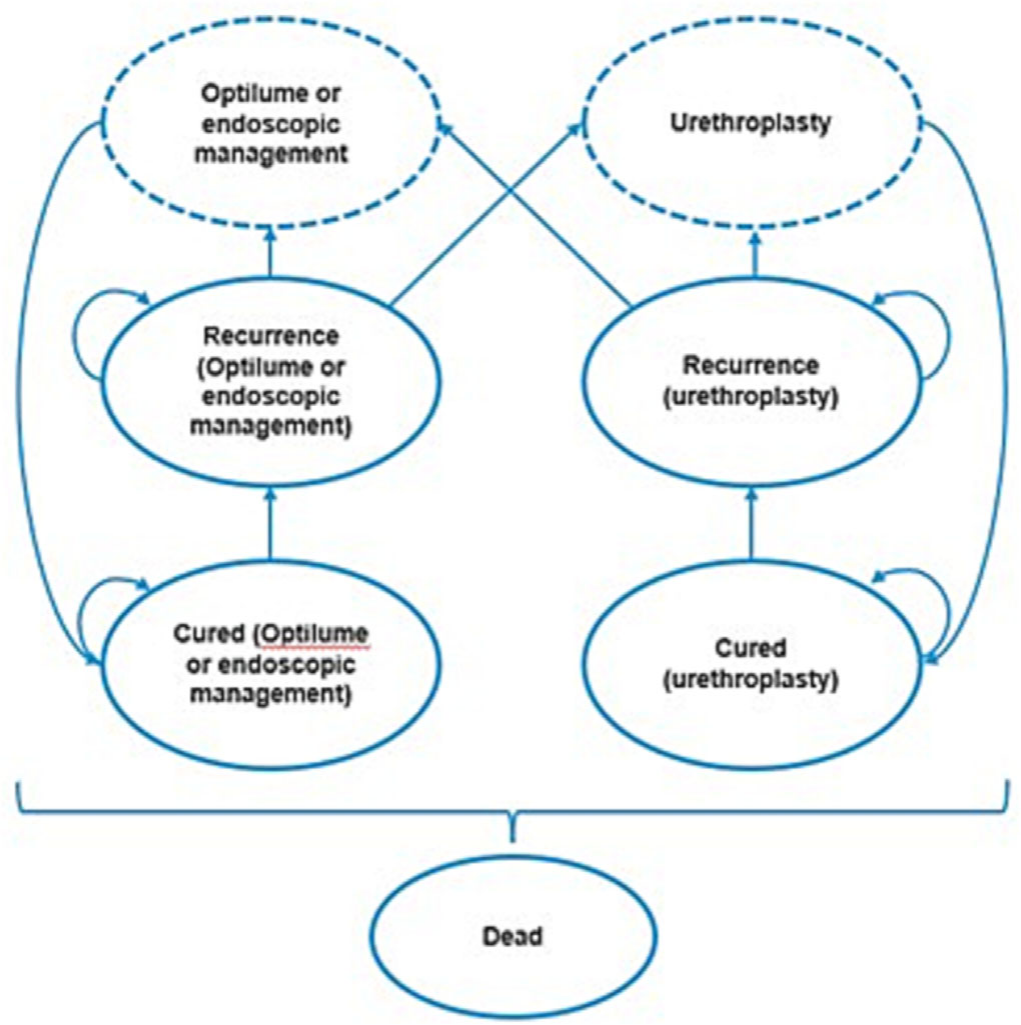
Patients entering the model underwent treatment with Optilume or endoscopic management. Patients transitioned to the treatment‐dependent cured health state after a one monthly‐cycle where they remained until experiencing a recurrence. A proportion of patients then transition into a recurrence health state that is dependent on the last treatment received. The number of cycles in which patients remain in the treatment‐dependent recurrence state is dependent on the median time to treatment following recurrence. A small proportion of recurrent patients are assumed to receive no treatment following stricture recurrence and will remain in this state for the remainder of the model time horizon or until death. The remainder of patients have a repeat procedure following recurrence, and patients can receive either a repeat endoscopic procedure or a urethroplasty procedure. Patients in the Optilume treatment arm given a repeat endoscopic procedure are assumed to have another Optilume treatment. Following retreatment, patients were assumed to transition to the cured health state, and any treatment failure was assumed to be captured by recurrence rates. At any point in the model time horizon, patients could transition and remain in the death health state.

The Markov model structure is shown in Figure 1. The mutually exclusive health states included

- Optilume or endoscopic management;
- urethroplasty;
- cured;
- recurrence; and
- death.

Patients could transition to a different health state or remain in their current health state after each monthly cycle. Patients could transition to the dead health state from any other health state and remained in the dead state.

The following assumptions were used in the modelling. Firstly, patients could remain in the recurrence health state for more than one cycle. This was based on literature showing that the time to treatment following recurrence is longer than 1 month^6^; the waiting time to treatment was also assumed to be equivalent between endoscopic management and Optilume. Another assumption made was that the efficacy of treatments was the same irrespective of whether it was an initial or repeat procedure. Evidence suggests that the efficacy of endoscopic management may diminish after the first treatment. However, the expected decrease of efficacy with each additional treatment is not clearly defined in the literature.^7^, ^8^ To support the assumption of keeping efficacy the same, the population that the model parameters are based on is heterogeneous, and patients may have had different numbers of previous endoscopic treatments at the time of recruitment into the study.

### 2.2 Inputs

The following input data were used to populate the model, and full details are in Table 1. Transition probabilities were derived for each health state based on data from the ROBUST III trial and OPEN RCT.^4^, ^6^ The monthly probability of recurrence after endoscopic management or Optilume was taken from the ROBUST III trial.^4^ The ROBUST III study utilised a subjective outcome at the 12‐month follow up, symptom recurrence without intervention. Responder rates at 12 months were based on International Prostate Symptom Score (IPSS) improvement of ≥30% without repeat intervention. The recurrence rate at 12 months was 88.1% for the standard of care arm and 26.9% for Optilume; these were then converted into monthly probabilities that could be used in the model. The reported outcomes of the ROBUST III were used for the transition probabilities as it directly compared Optilume and endoscopic management. The monthly probability of stricture recurrence with urethroplasty was taken from the OPEN RCT, using the outcome of recurrence at 24 months then converted to a monthly probability.^6^

**TABLE 1:** Model inputs.

Variable	Value	Source
Average patient starting age	59	Elliott et al., 2021a^4^
Discount rate (costs)	3.5%	NICE guidelines reference
Monthly probability of recurrence: endoscopic management	16.3%	Elliott et al., 2021a^4^
Monthly probability of recurrence: Optilume	2.6%	Elliott et al., 2021a^4^
Monthly probability of recurrence: urethroplasty	0.9%	Goulao et al., 2020^6^
Probability of treatment following stricture recurrence	90%	Goulao et al., 2020^6^
Proportion of patients treated with urethroplasty following recurrence after treatment with endoscopic management or Optilume	70%	Goulao et al., 2020^6^
Proportion of patients re‐treated with endoscopic management or Optilume following recurrence	30%	Goulao et al., 2020^6^
Proportion of patients re‐treated with urethroplasty following recurrence after urethroplasty	12%	Goulao et al., 2020^6^
Proportion of patients treated with endoscopic management or Optilume following recurrence after treatment with urethroplasty	88%	Goulao et al., 2020^6^
Median time to treatment following recurrence: endoscopic management and Optilume	47.5 days	Goulao et al., 2020^6^
Median time to treatment following recurrence: urethroplasty	90 days	Goulao et al., 2020^6^
Treatment cost: endoscopic management	£1196	NHS, 2021.^9^ LB55A Minor or intermediate urethra procedures 19 years and over. Weighted average.
Treatment cost: urethroplasty	£4761	NHS, 2021.^9^ LB29A Major Open Urethra Procedures, 19 years and over
Treatment cost (including device): Optilume	£1986	Provided by Optilume
Treatment cost (excluding device): Optilume	£635	Provided by Optilume
Cost of device: Optilume (excluding VAT)	£1350	Provided by Optilume
Cost of pre‐dilation: Optilume	£20.36	Provided by Optilume
Cost of adverse events: Optilume	£15.16	Calculation.^a^ NICE, 2021; NHS, 2021; Curtis & Burns, 2020^9^, ^10^, ^11^
Cost of adverse events: endoscopic management	£63.40	Calculation.^a^ NICE, 2021; NHS, 2021; Curtis & Burns, 2020^9^, ^10^, ^11^
Cost of adverse event: urethroplasty	£17.46	Calculation.^a^ NICE, 2021; NHS, 2021; Curtis & Burns, 2020^9^, ^10^, ^11^
Training cost (per patient): Optilume	£8.53	Provided by Optilume.
Cured health state cost (monthly)	£18.33	NHS, 2021.^9^ Outpatient 101 Urology service cost, assumption of two follow up appointments per year.
Total recurrence health state cost (monthly)	£44.74	NHS, 2021.^9^ Outpatient 101 Urology service cost, assumption of four follow up appointments per year.

^a^ View Table S1 for more details on how this was calculated.

The probability of having further treatment following a recurrence of a urethral stricture and the distribution of the different available treatments given was taken from the OPEN RCT.^6^ The study reported that 90% of patients received treatment following a recurrence; therefore, 10% of patients experiencing recurrence would remain untreated and, in the model, remain in the recurrent health state. The OPEN study is a recent UK RCT, which was judged to give the best estimate of how male patients with a urethral stricture are clinically managed in the United Kingdom.

Unit costs input into the model included those for the treatment specific procedures, staff training requirements and adverse events costs. The treatment cost of Optilume, excluding the device, is £635, which reflects that the Optilume procedure is likely to be undertaken in an outpatient setting. The adverse event cost was applied as a one off cost for each procedure; this was a weighted average cost calculated from the proportion of men experiencing the adverse event, up to 30 days post‐procedure, and the unit cost of treating the adverse event. Further details on the calculations used for the adverse events are in the supporting information. NHS reference costs, NICE BNF formulary prices and PSSRU unit costs were used when available at 2019/2020 prices.^9^, ^10^, ^11^

### 2.3 Scenario analysis

In scenario analysis, the effect of using urethroplasty as the comparator instead of endoscopic management was explored. To compare Optilume with urethroplasty, no inputs associated with Optilume were changed from the base case. The monthly probability of recurrence with urethroplasty was taken from the OPEN RCT, which stated a recurrence of 0.9%.^6^ The cost of urethroplasty (£4716) was taken from the NHS reference costs (10).

### 2.4 Sensitivity analysis

Sensitivity analyses were used to assess the level of confidence associated with the results of our economic evaluation. Both probabilistic sensitivity analysis (PSA) and deterministic sensitivity analysis (DSA) were conducted. A PSA was undertaken using 1000 iterations, because that was the number of iterations needed to produce stability in the results of the model. Distributions were fitted and used confidence intervals reported from the data sources. In the absence of data on the variability around the sampling distribution of mean values, the standard error was assumed equal to 25% of the mean. A summary of the distributions used for the DSA and PSA is available in Table S2.

## 3 RESULTS

The base case result followed a hypothetical cohort of men aged 59 with an anterior urethral male stricture. The mean cost per patient given Optilume, mean cost per patient given endoscopic management and the incremental difference are shown in Table 2. The total cost per person over a 5‐year time horizon was £9122 for endoscopic management and £6620 for Optilume. The result is a cost‐saving of £2502 when using Optilume when compared with endoscopic management in the NHS in England.

**TABLE 2:** Base case results, comparing the cost per patient of Optilume versus endoscopic management.

	Mean discounted cost per patient using Optilume (£)	Mean discounted cost per patient using endoscopic management (£)	Incremental difference in mean discounted cost per patient (£): Optilume vs. endoscopic management
Initial procedure cost (including device and adverse events)	£2001	£1259	£742
Repeat procedure costs (endoscopic)	£931	£1286	−£355
Repeat procedure costs (urethroplasty)	£2658	£5514	−£2856
Training costs	£9	£0	£9
Cost accrued in cured health state	£925	£860	£65
Costs accrued in recurrence health state	£97	£203	−£107
Total	£6620	£9122	−£2502

### 3.1 Scenario analysis

A scenario analysis demonstrates cost differences with the use of Optilume compared with urethroplasty. Table 3 shows the incremental cost difference between Optilume and urethroplasty, showing that the use of Optilume compared with urethroplasty results in a £243 cost saving per patient.

**TABLE 3:** Scenario analysis results, comparing the cost per patient of Optilume versus urethroplasty.

	Mean discounted cost per patient using Optilume (£)	Mean discounted cost per patient using urethroplasty (£)	Incremental difference in mean discounted cost per patient (£): Optilume vs. urethroplasty
Initial procedure cost (including device and adverse events)	£2001	£4779	−£2778
Repeat procedure costs (endoscopic)	£931	£544	£387
Repeat procedure costs (urethroplasty)	£2658	£543	£2115
Training costs	£9	£0	£9
Cost accrued in cured health state	£925	£956	−£31
Costs accrued in recurrence health state	£97	£42	£55
Total	£6620	£6863	−£243

### 3.2 Sensitivity analysis

**FIGURE 2:**
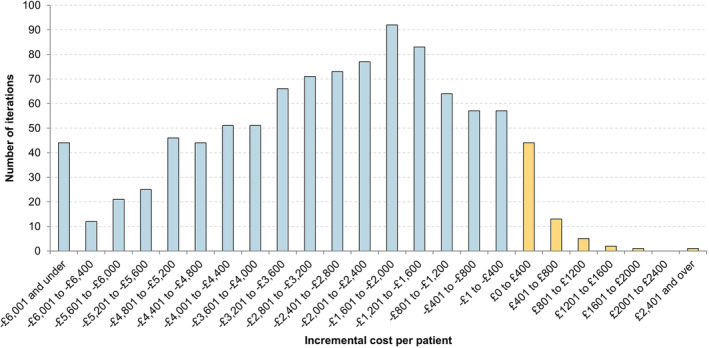
Histogram of probabilistic sensitivity analysis results of 1000 iterations.

The PSA demonstrates that the results are robust to joint parameter uncertainty. The majority of parameters were varied in the PSA by distributions based on confidence intervals reported in the literature. As Figure 2 shows, Optilume was cost saving in 93.4% of 1000 iterations.

**FIGURE 3:**
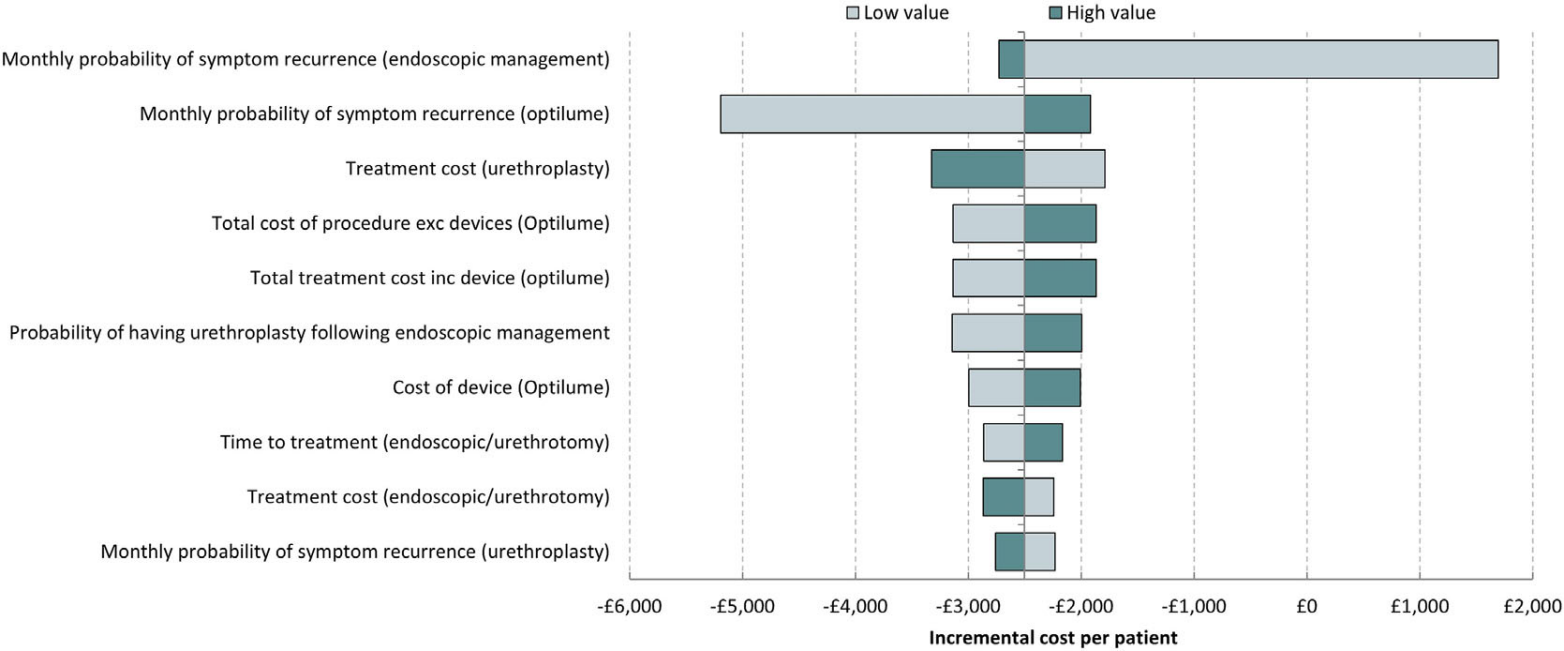
Tornado diagram of deterministic sensitivity analysis result.

The results of the DSA are presented in Figure 3, as a tornado diagram. The DSA shows that the monthly probabilities of symptom recurrence for endoscopic management and Optilume are the primary drivers of the incremental cost per patient. A table of the DSA results are available in Table S3.

## 4 DISCUSSION

The main findings of our analysis indicated that for the treatment of anterior male urethral stricture, the Optilume treatment is a cost‐saving procedure in comparison with endoscopic management, both in the base case and in over 90% of iterations of the PSA. In the base case, the Optilume results in an estimated cost saving of £2502 per patient if introduced in the NHS in England. The increase in the costs of initially using Optilume compared with endoscopic management is outweighed by the costs saved from a reduction in procedure recurrence. In the scenario analysis looking at urethroplasty as a comparator, Optilume was cost saving with an estimated saving of £243 per person.

The main strength associated with this analysis is the use of robust model inputs adopted from publicly available data sources. Costs were taken from the BNF and NHS reference costs, sources that are widely adopted for economic evaluations in England.^9^, ^10^ The effectiveness of Optilume was modelled based on data from the pivotal, phase III, single‐blind, randomised controlled study ROBUST III.^4^ The ROBUST III consisted of 127 subjects. From the ROBUST III, our analyses estimated the probability of stricture recurrence and safety of Optilume compared with endoscopic management. The results of this trial are supported by a single arm study of Optilume looking over a longer 3‐year time period, the ROBUST I.^12^ These key model assumptions were then extensively explored via sensitivity analyses. The PSA and DSA showed that the model results are robust to plausible changes in input parameters.

There were some limitations identified in our analyses. The ROBUST III trial, used for treatment recurrence rates of Optilume and endoscopic management, was conducted in the United States and reported considerably higher recurrence rates than the United Kingdom based OPEN trial. The monthly recurrence probabilities reported in ROBUST III for endoscopic management and Optilume are 16.3% and 2.6%, respectively, in the OPEN RCT they are 1.9% and 0.5%.^4^, ^6^ The ROBUST III trial was determined to be a better data source than the OPEN RCT as the given rates are generally in line with those reported in other studies evaluating treatments for stricture recurrence.^7^, ^8^, ^13^ It is expected that the ROBUST III data are generalisable to England. However, it is acknowledged that high recurrence rates may reflect that a harder to treat population was used due to the trials strict inclusion criteria.

Another limitation in our analyses was the lack of robust data to inform the comparison between Optilume and urethroplasty. Urethroplasty was included in the scenario analysis as the comparator to Optilume. There was not any head to head data available that directly compared the effectiveness of both treatments, in the absence of data an indirect comparison was made to estimate the relative risk of recurrence between the two treatment options. It was determined that this scenario was still important to run, despite the data limitations, due to urethroplasty being an alternative treatment provided by the NHS in England.

A review of the literature, at time of analyses, found no previous economic evaluations of Optilume. The Shen et al. study conducted an economic evaluation alongside the OPEN RCT which compared the cost‐effectiveness of urethroplasty with endoscopic management.^14^ The paper reported costs of £6553 and £8026 for urethrotomy and urethroplasty, respectively. Comparisons between this evaluation and our analyses are limited as they used a 10‐year time horizon, which means results are not directly comparable with our 5‐year outcomes.

Future research in this area could be conducted into the monthly probability of recurrence taken from England with a wider population group, looking specifically at Optilume. A micro‐costing study into the NHS England cost of endoscopic procedures may also provide more accurate inputs for costs. By doing future research, this could provide more accurate estimates for the model. However, our results were robust to variations in the inputs, and new estimates are unlikely to change the direction of the results.

## 5 CONCLUSION

In conclusion, the findings of this analysis support the use of Optilume urethral drug‐coated balloon as an alternative to the standard of care endoscopic management in the treatment of men with recurrent anterior male urethral stricture in England. This was shown in the base case scenario and in the results of the deterministic and probabilistic sensitivity analysis.

## AUTHOR CONTRIBUTIONS

*Substantial contributions to study methodology and analysis*: Judith Shore, Laura Kelly and Hayden Holmes. *Writing, reviewing and editing the article*: Laura Kelly, Hayden Holmes, James Wright, and Craig Patrick.

## DISCLOSURE OF INTEREST

There are no competing interests for Laura Kelly, Judith Shore or Hayden Holmes. James Wright and Craig Patrick are all employees of Laborie and receive honorarium from the organisation.

## Supporting information


**Table S1:** Adverse events costing and prevalence for Optilume, endoscopic management and urethroplasty.
**Table S2:** Distributions used for deterministic and probabilistic sensitivity analysis.
**Table S3:** DSA results
