# Supplementary material for: Economic evaluation of Optilume, a drug‐coated balloon for recurrent anterior male urethral stricture
Source: BJUI Compass. 2023 Apr 10;4(4):430–6. doi: 10.1002/bco2.241 (PMC10268567; doi:10.1002/bco2.241)
Supplement: Supplementary file 1 — Table S1: Adverse events costing and prevalence for Optilume, endoscopic management and urethroplasty. Table S2: Distributions used for deterministic and probabilistic sensitivity analysis. Table S3: DSA results [file BCO2-4-430-s001.docx]

Suppl Tab 1: Adverse events costing and prevalence for Optilume, endoscopic management and urethroplasty

|  | **Unit cost** | **Optilume** | **Endoscopic management** | **Urethroplasty** |
| --- | --- | --- | --- | --- |
| **Haematuria** | £33  (GP appointment (1)) | 0.0% (2) | 0.0%(2) | 2.0%(3) |
| **Urinary tract infection** | £43  (GP appointment (1);  Average antibiotic cost of Nitrofurantoin and Trimethoprim(4);  Urinalysis (5)) | 7.6% (2) | 8.3%(2) | 3.1%(3) |
| **Wound infection** | £107  (GP appointment (1);  Average antibiotic cost of Flucloxacillin (4)) | 0.0%* | 1.0% (3) | 2.0%(3) |
| **Readmission to hospital** | £434  (Weighted average non-elective short stay  LB57C/ LB57D Urethral Disorders with/without Interventions (6)) | 0.0%* | 0.0%* | 3.1% (3) |
| **Urinary retention (requiring emergency intervention)** | £941  (Accident and emergency. LB55A Minor or intermediate, urethra procedures (6)) | 1.3% (2) | 6.3% (2) | 0.0%* |

*Assumption

Suppl Tab 2: Distributions used for deterministic and probabilistic sensitivity analysis

| **Parameter** | **Base case value** | **Range and source used for DSA** | **Range and source used for PSA** |
| --- | --- | --- | --- |
| Average patient starting age | 59.42 | Lower and upper bound 44.56 to 74.27  (Range taken from ROBUST III trial (2)) | Not varied in PSA |
| Discount rate: costs | 3.5% | Lower and upper bound 2% to 4%  (Assumption of a plausible range) |  |
| Monthly probability of recurrence: endoscopic management | 16.3% | Lower and upper bound 1.94% to 20.3%  (Lower from OPEN RCT, upper is 25% variation from mean(3))  Wider variation explored in two-way SA, varied between 1% and 21% | Distribution Beta (Alpha 7.64, Beta 39.36)  ROBUST III (2) |
| Monthly probability of recurrence: Optilume | 2.6% | Lower and upper bound 0.5% to 3.25%  (Lower based on OPEN RCT and combined with RR estimated from ROBUST III, upper is 25% variation from mean (2, 3))  Wider variation explored in two-way SA, varied between 0.2% and 4.2%. | Distribution Beta  (Alpha 2.01, Beta 75.99)  OPEN RCT (Pickard, 2020) (3) |
| Monthly probability of recurrence: urethroplasty | 0.95% | Lower and upper bound 0.71% to 1.18%  (Based on 25% variation from the mean (3))  Wider variation explored in two-way SA, varied between 0.6% and 1.6% | Distribution Beta  (Alpha 0.88, Beta 92.12)  OPEN RCT (Pickard, 2020) (3) |
| Probability of treatment following stricture recurrence | 90% | Lower and upper bound 67.5% to 100%  (Based on 25% variation from the mean) | Distribution Beta  (Standard error 0.2, Alpha 1.13, Beta 0.13) OPEN RCT (Pickard, 2020) |
| Proportion of patients treated with urethroplasty following recurrence after treatment with endoscopic management or Optilume | 70% | Lower and upper bound 52.5% to 87.5%  (Based on 25% variation from the mean) | Distribution Beta  (Standard error 0.08, Alpha 22.27, Beta 9.54)  OPEN RCT (Pickard, 2020) |
| Proportion of patients re-treated with urethroplasty following recurrence after urethroplasty | 12% | Lower and upper bound 9% to 15%  (Based on 25% variation from the mean) | Distribution Beta  (Standard error 0.12, Alpha 0.76, Beta 5.57)  OPEN RCT (Pickard, 2020) |
| Median time to treatment following recurrence: endoscopic management and Optilume | 47.5 days | Lower and upper bound 28 to 88 days  (Range stated in OPEN RCT) | Distribution Logormal  (Standard deviation 0.8, standard error on log scale 0.08) Estimated using log of IQR divided by 1.35 due to mean not being reported  (Pickard, 2020) |
| Median time to treatment following recurrence: urethroplasty | 90 days | Lower and upper bound 53 to 157  (Range stated in OPEN RCT) | Distribution Lognormal  Standard deviation 0.8, standard error on log scale 0.09) Estimated using log of IQR divided by 1.35 due to mean not being reported(Pickard, 2020) |
| Treatment cost: endoscopic management | £1,196 | Lower and upper bound £1,067 to £1,376  (Lower based on NICE MIB and updated to most recent NHS reference costs, upper from OPEN RCT)  Wider variation explored in two-way SA, varied between £900 and £1,900. | Distribution Gamma (Alpha 100, Beta 12)  Standard error of 10% assumed |
| Treatment cost: urethroplasty | £4,761 | Lower and upper bound £3,571 to £6,139  (Lower is 25% variation from mean, higher from OPEN RCT(3)) | Distribution Gamma (Alpha 25, Beta 190)  Standard error of 20% assumed |
| Treatment cost (including device): Optilume | £1,986 | Lower and upper bound £1,554 to £2,418  (Lower based on assumption of an outpatient procedure, upper from assumption of a day case procedure(6)) | Distribution Gamma (Alpha 100, Beta 20)  Standard error of 10% assumed |
| Treatment cost (excluding device): Optilume | £635 | Lower and upper bound £203 to £1,067  (Lower based on assumption of an outpatient procedure, upper from assumption of a day case procedure(6))  Wider variation explored in two-way SA, varied between £200 and £1,200. | Not varied within PSA, all varied as part of total treatment cost above. |
| Cost of device: Optilume | £1,350 | Lower and upper bound £1,012.50 to £1,687.50  (Based on 25% variation from the mean) |  |
| Cost of predilation: Optilume | £20.36 | Lower and upper bound £15.27 to £25.45  (Based on 25% variation from the mean) |  |
| Cost of adverse events: Optilume | £15.16 | Lower and upper bound £11.61 to £19.35  (Based on assumption of 25% more or fewer adverse events) | Distribution Gamma (Alpha 25, Beta 1)  Standard error of 20% assumed |
| Cost of adverse events: endoscopic management | £63.40 | Lower and upper bound £47.92 to £79.88  (Based on assumption of 25% more or fewer adverse events) | Distribution Gamma (Alpha 25, Beta 3) Standard error of 20% assumed |
| Cost of adverse event: urethroplasty | £17.46 | Lower and upper bound £13.19 to £24.13  (Based on assumption of 25% more or fewer adverse events) | Distribution Gamma (Alpha 25, Beta 3) Standard error of 20% assumed |
| Training cost (per patient): Optilume | £8.53 | Lower and upper bound £6.40 to £10.66  (Based on 25% variation from the mean) | Distribution Gamma (Alpha 25, Beta 0)  Standard error of 20% assumed |
| Cured health state cost (monthly) | £18.33 | Lower and upper bound £9 to £37  (Based on assumption of changing the number of follow up appointments to 1 for lower and 4 for upper) | Distribution Gamma (Alpha 25, Beta 1)  Standard error of 20% assumed |
| Total recurrence health state cost (monthly) | £44.74 | Lower and upper bound £34 to £56  (Based on 25% variation from the mean) | Distribution Gamma (Alpha 25, Beta 2)  Standard error of 20% assumed |

1. Curtis L, Burns A. Unit Costs of Health and Social Care 2020. Personal Social Services Research Unit, University of Kent, Canterbury; 2020.

2. Elliott S, Coutinho K, Robertson KJ, D'Anna R, Chevli K, Carrier S, et al. One-Year Results for the ROBUST III Randomized Controlled Trial Evaluating the Optilume Drug-Coated Balloon for Anterior Urethral Strictures. The Journal of urology. 2021:10.1097/JU. 0000000000002346.

3. Pickard R, Goulao B, Carnell S, Shen J, MacLennan G, Norrie J, et al. Open urethroplasty versus endoscopic urethrotomy for recurrent urethral stricture in men: the OPEN RCT. Health Technology Assessment (Winchester, England). 2020;24(61):1.

4. National Institute for Health and Care Excellence. British National Formulary 2021 [Available from: <https://bnf.nice.org.uk/>.

5. National Institute for Health and Care Excellence. Routine preoperative tests for elective surgery. <https://www.nice.org.uk/guidance/ng45>; 2016.

6. National Health Service. 2019/20 National Cost Collection data Version 2. <https://www.england.nhs.uk/national-cost-collection/2021>.

Suppl Tab 3: DSA results

| Parameter | Base case value | Low value | | High value | |
| --- | --- | --- | --- | --- | --- |
|  |  | Value | Outcome | Value | Outcome |
| Discount rate | 3.5% | 2.0% | **-£2,543** | 4.0% | **-£2,488** |
| Average patient starting age | 59.4 | 43.00 | **-£2,518** | 75.00 | **-£2,428** |
| Monthly probability of symptom recurrence (optilume) | 2.6% | 0.5% | **-£5,194** | 3.3% | **-£1,918** |
| Monthly probability of symptom recurrence (endoscopic management) | 16.3% | 1.9% | **£1,694** | 20.3% | **-£2,728** |
| Monthly probability of symptom recurrence (urethroplasty) | 0.9% | 0.7% | **-£2,233** | 1.2% | **-£2,760** |
| Probability of having treatment following recurrence of symptoms | 90.0% | 67.5% | **-£2,465** | 100.0% | **-£2,501** |
| Probability of having urethroplasty following endoscopic management | 70.0% | 52.5% | **-£3,141** | 87.5% | **-£1,995** |
| Probability of having urethroplasty following urethroplasty | 12.0% | 9.0% | **-£2,506** | 15.0% | **-£2,497** |
| Time to treatment (endoscopic/urethrotomy) | 47.5 | 28.00 | **-£2,864** | 88.00 | **-£2,165** |
| Time to treatment (urethroplasty) | 90.0 | 53.00 | **-£2,298** | 157.00 | **-£2,759** |
| Treatment cost (endoscopic/urethrotomy) | £1,195.78 | £1,067.00 | **-£2,242** | £1,376.00 | **-£2,866** |
| Treatment cost (urethroplasty) | £4,761.47 | £3,571.00 | **-£1,790** | £6,139.00 | **-£3,325** |
| Total treatment cost inc device (optilume) | £1,986.00 | £1,554.00 | **-£3,135** | £2,418.00 | **-£1,869** |
| Total cost of procedure exc devices (Optilume) | £634.98 | £203.00 | **-£3,135** | £1,067.00 | **-£1,869** |
| Cost of device (Optilume) | £1,350.00 | £1,012.50 | **-£2,996** | £1,687.50 | **-£2,007** |
| Cost of predilation (Optilume) | £20.36 | £15.27 | **-£2,502** | £25.45 | **-£2,501** |
| Cost of adverse event (Optilume) | £15.16 | £11.61 | **-£2,507** | £19.35 | **-£2,496** |
| Cost of adverse event (endoscopic/urethrotomy) | £63.40 | £47.93 | **-£2,471** | £79.88 | **-£2,535** |
| Cost of adverse event (urethroplasty) | £17.46 | £13.19 | **-£2,499** | £24.13 | **-£2,506** |
| Training cost (per patient) | £8.53 | £6.40 | **-£2,504** | £10.66 | **-£2,500** |
| Cured health state cost (monthly) | £18.33 | £9.00 | **-£2,535** | £37.00 | **-£2,436** |
| Total recurrence health state costs (monthly) | £44.74 | £33.55 | **-£2,475** | £55.92 | **-£2,528** |
